# Comments regarding the recent publication by Abou Chedid et al. 2023 Comparative study of two different computer-controlled local anesthesia injection systems in children: a randomized clinical trial

**DOI:** 10.1007/s40368-024-00969-5

**Published:** 2024-12-04

**Authors:** M. N. Hochman

**Affiliations:** 1 Former Clinical Associate Professor at School of Dental Surgery, Department of Implants Dentistry and Orthodontics, New York University, New York, NY USA; 2 Clinical Consultant, Milestone Scientific, Inc, Rosedale, NJ USA; 3 Periodontics / Implant Dentistry / Orthodontics, Synergistic Dentistry of New York, New York, NY USA

Dear Editor in Chief,

Computer-controlled Local Anesthetic Delivery (CCLAD) has been shown to be an effective tool in reducing subjective pain perception during the administration of local anesthetics to dental patients. It has been especially beneficial in the treatment of pediatric dental patients by showing both, a substantial reduction in subjective pain perception, as well as a marked reduction in pain disruptive behavior in this dental population. (Garret-Bernardin et al. 2017; Giannetti et al. 2018) One of the earliest pioneers in CCLAD research in children was Dr. Malka Ashkenazi. Her team demonstrated the benefits and safety of performing intrasucular (a.k.a. Intraligamentary) injection anesthesia on deciduous teeth in the pediatric dental patient. The first CCLAD instrument introduced to the dental profession was *The Wand*^*®*^ dental injection system from Milestone Scientific, Inc. Livingston, NJ in 1997. I was the principal investigator of the research team that published the first clinical comparison study in humans which was published in the New York State Dental Journal in 1997. (Hochman et al. 1997).

In 2007, Milestone Scientific, introduced the next generation CCLAD instrument: The Wand/STA System. The technology behind CCLAD that makes it capable of reducing/eliminating pain during an injection is directly related to precisely controlling multiple variables of the injection technique that cannot be controlled with a manual hand-held conventional syringe. The two most important variables are: (1) fluid flow-rate and (2) fluid-pressure generated during a subcutaneous injection. The precise control of these two variables, irrespective of tissue density, is of critical importance in achieving pain reduction and the elimination of pain. This was first described in detail in 2006. (Hochman et al. 2006) Since this first study, there have been numerous authors that have emphasized that using the slow-flow rate (i.e., 0.005 mL/sec) is an essential component to achieving pain reduction/elimination during injection. (Flisfisch et al. 2021; Perugia et al. 2017) In addition to these variables, needle manipulation and tactile control of the needle are also understood as key elements to the reduction of pain perception when performing a dental injection.

In the March 2023 issue of this journal a comparison between two CCLAD Systems was reported by Abou Chedid and co-workers in an article titled ‘**Comparative study of two different computer-controlled local anesthesia injection systems in children: a randomized clinical trial**’ claiming to be the first study to compare two CCLAD systems. (Abou Chedid et al. 2023) After a thorough review of the article a major deficiency was identified in the study design, specifically, an inconsistency that would explain the reported results. It appears that the methodology used for testing the STA instrument against the Calaject may have had a critical design flaw, leading to results that unfairly represented the STA instrument. This position is outlined in the following material.

**The authors provided the following description in the “Materials/Methods" of their article:** *“For the STA, the normal mode was selected for buccal infiltration. 1/4 of the injection was done with the ControlFlo rate (0.005 ml/s), when the ‘cruise’ sound was emitted by the machine, stipulating that the needle was positioned correctly and that it could be advanced in the tissue, the rate was switched to RapidFlo (0.03 ml/s). For Calaject, program 2 was selected. The injection was done at 0.006 ml/s for 10 s, and then a rapid flow of 0.03 ml/s until the carpule was finished. The entire injection was completed in approximately 100 s for both devices.”*

## The points of concern are as follows:

Contradiction and Inconsistency in Study Design: The authors stated that 1/4 of the injection was completed at the ControlFlo rate (0.005 ml/s) before switching to the RapidFlo rate (0.03 ml/s) upon the “cruise” sound announced by the STA instrument. The “cruise” spoken announcement occurs at 4 s from the start of the injection and is meant to allow the user to release the foot-control for continued administration and not meant to signify switching to the faster flow-rate. Switching from the slow flow-rate to the more rapid flow-rate only after 4 s would explain the greater discomfort when compared to the Calaject, which was given a full 10 s at the slow flow-rate (0.006 ml/s) before automatically changing to the faster flow-rate (0.03 ml/s). This difference may lead to bias against the STA instrument. Switching to the RapidFlo flow-rate prematurely results in a clinical situation in which insufficient anesthetic drug was deposited prior to switching flow-rates and would likely result in a more uncomfortable injection for patients. A larger amount of drug is required before switching from a slow flow-rate to a faster flow-rate for injection using any CCLAD instrument.

Discrepancy with Previous Studies and Clinical Recommendations: Numerous studies have documented that the STA’s ControlFlo rate (0.005 ml/s) produces a virtually painless experience for patients when used properly, even for difficult injections. (Baghlaf et al. 2018; Helmy et al. 2022) In this current study, the Calaject instrument had used a slow flow-rate (0.006 ml/s) and was given 2.5 times the amount of time at the slow flow-rate before switching. In addition, the Calaject injections were provided a larger volume of anesthetic drug to be present before switching from the slow flow-rate to the faster flow-rate. This discrepancy likely contributed to the increased stimulation reported within the Wand/STA patient group. This is another contributing factor that may have resulted in biasing the results of this study.

Non-Compliance with STA System Use-of-Instructions Manual: According to the owner’s manual from the STA Injection System on page 12, of “BASIC OPERATION” it states, and I quote, “*Important: ControlFlo should be used at the beginning of ALL injection techniques. It provides a controlled and safe administration that normally results in little or no discomfort. Once initial “numbness” has occurred you may decide to switch to a more rapid rate, i.e., RapidFlo or TurboFlo during infiltration injections and Inferior Alveolar block injections. Typically ¼ of the cartridge should be administered using the ControlFlo rate before switching to a more rapid rate of delivery*” the recommendation of using the ControlFlo flow-rate (0.005 ml/s) for the first quarter of the cartridge (0.4 mL) before switching to a more rapid rate of delivery was not adhered to in this study design. As this typically requires approximately 110 s and the authors stated they completed the entire injection in 100 s. It appears the study performed switched between ControlFlo flow-rate to RapidFlo at 4 s, contradicting the manual’s guidelines and recommendations. This inconsistency highlights a potential significant flaw in the study design which may have led to misleading results.

The objective outlined in this study was to compare two different CCLAD systems. However, to achieve this within a study design it is imperative to adhere to the manufacturer’s recommendations and instructions-for-use otherwise unintended bias may appear within the data and create greater confusion instead of greater clarity. Also, it would have been beneficial if an in-depth discussion was provided as to how the variables of flow-rate, the amount of drug administered at each flow-rate, time administered for each flow-rate, and the total drug volume administered impacted the results for each system used in this study. As CCLAD systems have become an important part of the armamentarium for many clinicians today, clinical research on this topic can help to identify the differences and the benefits of these systems. I respectfully provided my comments to this journal and to the authors of this article in the spirit of clarifying several important limitations that were identified and may have impacted the results reported in this study. Thank you for opportunity to share my perspective.

Sincerely yours,

Mark N. Hochman, DDS.
